# Mechanically assisted intra-arterial thrombolysis in acute cerebral infarction

**DOI:** 10.3892/etm.2013.990

**Published:** 2013-03-06

**Authors:** HUI-XIAO WANG, YI-JIN SHEN, SHU-JUN YE, YONG-KANG XU, JIAN-PIN ZHANG, ZHOU LU

**Affiliations:** Department of Neurosurgery, Affiliated Yinzhou Hospital, College of Medicine, Ningbo University, Ningbo, Zhejiang 315040, P.R. China

**Keywords:** acute cerebral infarction, mechanically assisted thrombolysis, bleeding complication, recanalization rate

## Abstract

The aim of this study was to assess the clinical efficacy and safety of mechanically assisted thrombolysis in the treatment of acute cerebral infarction. Mechanically assisted intra-arterial urokinase thrombolysis was conducted on 28 patients with acute cerebral infarction with a disease onset time of 90–450 min. The maximum level of urokinase was 1,150,000 units. Thrombus disruption with a microwire, retrieval with a microcatheter and stent-assisted revascularization were performed. The recanalization rate, bleeding complications and modified Rankin scale (mRS) score were observed within 3 months of surgery. Our results showed that mechanically assisted thrombolysis was successfully conducted on 23 patients, with a recanalization rate of 82.1% (23/28), average recanalization time of 65.22 min and mRS score ≤3.5. Five cases of recanalization were invalid, including 2 cases of mortality, 1 case with an mRS score of 4 and 2 cases with an mRS score ≤3. In the recanalization group, the mechanically assisted thrombolysis did not increase the number of bleeding complications. Our study demonstrated that the safety of mechanically assisted thrombolysis for the treatment of acute cerebral infarction is equivalent to that of simple intra-arterial thrombolysis, but that the former has a higher efficiency. Mechanically assisted thrombolysis is able to reduce the urokinase dosage and recanalization time, and increase the recanalization rate.

## Introduction

There are 110 individuals with cerebral infarction per 100,000 population in China, accounting for 60–80% of patients with stroke (1). In 75% of the cases, the cerebral infarction is caused by local cerebrovascular occlusion due to acute thrombosis formation or thrombosis metastasis from other sites. With the development of neurological and thrombolytic research, thrombolytic therapy has become the most effective treatment for reducing the infarction area and disability rate, particularly in patients with moderate or severe nervous disorders. Aggressive and reasonable thrombolytic technologies may be used in hyper-acute and acute cerebral infarction for restoring the blood supply in the ischemic penumbra. This rescues the nerve cells with reversible damage and thus reduces the mortality and disability rate of patients,. which is important for the patient, family and society. It has been observed that if intravenous thrombolysis using recombinant tissue-type plasminogen activator (rTPA) is conducted within 3 h after ischemic stroke onset, the risks of fatality and severe disability are significantly reduced, with great improvements in the quality of life of survivors (2). As shown in clinical studies, intra-arterial thrombolysis has a more reliable efficacy, with a longer therapeutic time window (3–5). However, the recanalization time for intravenous and intra-arterial thrombolysis is at least 1–2 h (6–8), rarely <1 h. An exception has been reported by Farkas *et al*(9), in which the average recanalization time was 54 min for 17 patients treated with intra-arterial rTPA thrombolysis. In addition, the recanalization rate of intra-arterial thrombolysis remains unsatisfactory.

Further reduction of the recanalization time relies on mechanically assisted thrombolysis, i.e., the combination of thrombolytic injection and mechanical thrombus disruption or removal. The latter may further reduce the thrombolytic dosage and increase the contact area of the thrombolytic agent with the thrombus, resulting in safer intra-arterial thrombolysis and a higher recanalization rate (10–14). Therefore, an understanding of the factors influencing recanalization and the identification of the most favorable mechanically assisted thrombolysis methods are particularly important. In this study, mechanically assisted intra-arterial urokinase thrombolysis was conducted in 28 patients with acute cerebral infarction between January 2009 and October 2012. The clinical efficacy and safety of mechanically assisted thrombolysis in the treatment of acute cerebral infarction were assessed.

## Patients and methods

### Patients

According to the cerebral infarction diagnosis standard (Fourth National Cerebrovascular Diseases Conference, China) (15) and Guideline of Cerebrovascular Disease Prevention and Treatment in China (People’s Health Publishing House, 2007) (16), 28 patients (20 males and 8 females) diagnosed with acute cerebral infarction between January 2009 and October 2012 were enrolled in this study. The patients were aged 33–78 years old, with an average age of 57.6 years. There were 23 cases with a thrombus in the internal carotid artery system and 5 cases with a thrombus in the vertebral-basilar artery system. A total of 20 cases were treated within 3–6 h after thrombosis onset and 8 cases were treated within 6–8 h after thrombosis onset. The cases of combined vascular stenosis (>50%), hypertension, diabetes, atrial fibrillation, transient ischemia attack (TIA) and cerebral infarction history were 7 (25.0%), 16 (57.1%), 6 (21.4%), 3 (10.7%), 8 (28.6%) and 3 (10.7%), respectively (Table II). The study was supported by Ningbo Medical Technology project (No. 2006058) has been approved by the ethics committee of the Affiliated Yinzhou Hospital, Ningbo, China. Informed consent was obtained from the patient or the patient’s family.

### Mechanically assisted thrombolysis

The preoperative routine examinations, including CT scanning, blood coagulation test, blood sugar test and electrocardiogram were performed on patients prior to thrombolysis, and the routine preoperative preparation was conducted. The cerebral angiography and arterial thrombolysis were then conducted immediately. The CT scanning results were ready within 24 h. The time between emergency visit and treatment initiation was ≤45 min.

**Figure 1:**
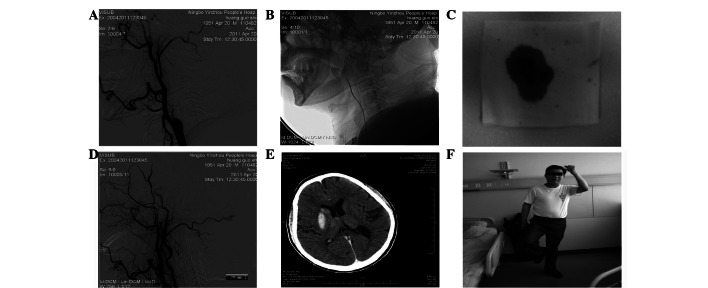
Therapeutic process of mechanically assisted intra-arterial thrombolysis. (A) Occlusion in the right internal carotid artery; (B) catheter insertion into the thrombus guided by microwire; (C) thrombus retrieved by a 5-ml injector; (D) internal carotid artery tandem stenosis after recanalization (second stent); (E) bleeding after recanalization (second stent); (F) excellent restoration two weeks later.

**Figure 2:**
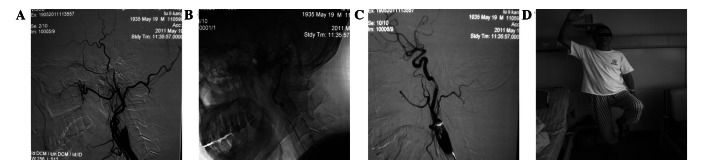
Therapeutic process of mechanically assisted intra-arterial thrombolysis. (A) Occlusion in the left internal carotid artery; (B) contrast agent retention following mechanical disruption; (C) following continued use of urokinase; (D) excellent restoration one week later.

Systemic heparinization was performed following a puncture to the femoral artery using Seldinger technology. The aortic arch and cerebral angiography were conducted under local anesthesia to determine the infarction site (the angiography was often conducted on the aortic arch and suspected sites according to clinical indicators to save time). After the infarction site was confirmed, heparin (40 IU/kg) was administered to the patient. For patients with thrombus formation in the internal carotid artery or vertebral-basilar artery, the thrombosis treatments were as follows: i) for 5 patients with complete vascular occlusion, a 0.035-inch guidewire and angiography catheter were used for thrombus disruption. After the guidewire had passed the thrombus site, the thrombolysis was conducted and the thrombus was removed using a 5-ml injector (Figs. 1 and 2). ii) For patients with severe internal carotid artery stenosis (hemodynamic infarction), a dose of 400,000 units of urokinase was administered for 1 h of thrombolysis. If the stenosis site did not change, the first stent-assisted revascularization was conducted (1 case). If the stenosis was eased, the thrombolysis was continued until the total amount of urokinase reached 1,000,000 units. Then 2–3 weeks of routine anticoagulation therapy was performed, followed by re-examination. The second stent-assisted revascularization was conducted on 1 case.

**Figure 3:**
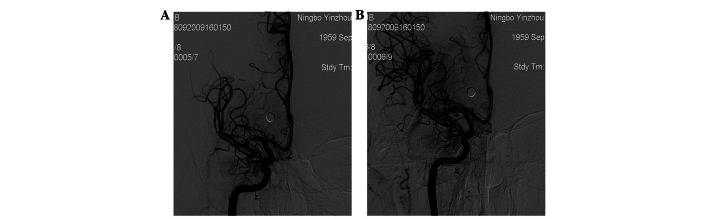
Recanalization was performed on the remaining thrombus, and the stent-assisted revascularization was conducted for vascular stenosis (≥50%) in the first stent after thrombolysis. (A) Right middle cerebral artery stenosis following recanalization; (B) application of the first stent.

**Figure 4:**
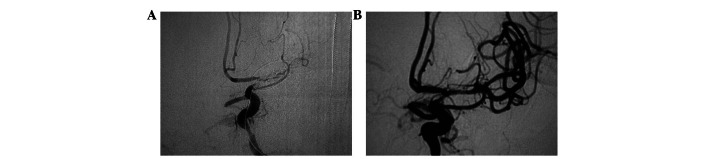
Recanalization was performed on the remaining thrombus, and the stent-assisted revascularization was conducted for vascular stenosis (≥50%) in the second stent after thrombolysis. (A) Left middle cerebral artery occlusion (guidewire disruption); (B) distal internal carotid artery stenosis following recanalization (the second stent).

For patients with a thrombus in the middle cerebral artery, the thrombosis treatments were as follows: i) for complete vascular occlusion cases, retrieval with a guidewire (J-type rotation) or repetitive interpenetration with a guidewire or catheter were conducted, followed by thrombolysis. ii) 400,000 units of urokinase were intra-arterially perfused within 0.5 h, followed by angiography. Urokinase (150,000 units) was then arterially perfused and angiographic re-examination was conducted every 10 min until the total urokinase amount reached 1,000,000 U. Following thrombolysis, recanalization was performed on the remaining thrombus and the stent-assisted revascularization was conducted for vascular stenosis ≥50% (the first stent, 1 case, Fig. 3; the second stent, 2 cases, Fig. 4).

During treatment, the maximum urokinase dosage was 1,150,000 U. Following thrombolysis, the arterial sheath was retained for 1 h. The intraoperative and postoperative cardiogram, blood pressure and blood oxygen saturation were continuously monitored. Cranial CT scans were performed immediately after surgery and 24 h later to determine the extent of intracranial hemorrhaging. At 24 h after thrombolysis, Bayaspirin (300 mg/day) was orally administered. For patients with stent-assisted revascularization, Bayaspirin (300 mg/day) and Clopidogrel (75 mg/day) were used together for 3 months, followed by only Baspirin (100 mg/day) for a prolonged period of time.

### Evaluation of treatment efficacy

The treatment efficacy was evaluated according to the indices as follows: i) Thrombolysis in Myocardial Infarction (TIMI) grades of recanalization in imaging manifestation: grade 0, no blood perfusion of the distal occluded artery; grade 1, partial passage of contrast agent, partial filling of the distal stenotic artery; grade 2, complete filling of the distal stenotic artery, slow development and elimination of contrast agent; and grade 3, rapid filling and elimination in the distal stenotic artery, same with normal artery. In this study, TIMI grades 2 and 3 were defined as recanalization. ii) Evaluation standard from National Institutes of Health stroke scale (NIHSS; perioperative), iii) bleeding complications and iv) modified Rankin scale (mRS) score (postoperative 3 months; Table I).

### Statistical analysis

Data were expressed as mean ± SD. Statistical analysis was performed using SPSS 17.0 statistical software (SPSS, Chicago, IL, USA). A Student’s t-test was used for measurement data. For small-sized samples, the F-statistic was calculated according to the Levene test (P=0.1). Then t-test and Cochran and Cox t-tests were performed for homogeneity (P>0.1) and heterogeneity of variance (P<0.1), respectively. A Chi-square test was conducted for counted data. P<0.05 was considered to indicate a statistically significant result.

## Results

The mechanically assisted thrombolysis was successfully conducted on 23 patients, with a recanalization rate of 82.1% (23/28) and an average recanalization time of 65.22 min. The patient age, disease onset time, urokinase dosage and preoperative NIHSS score in the recanalization group (23 cases) and non-recanalization group (5 cases) are shown in Table II. The results show that there were significant differences in disease onset time and age between the two groups, with no statistical difference between the urokinase dosage and preoperative NIHSS scores.

The indices in patients with bleeding complications (14 cases) and without bleeding complications (9 cases) are shown in Table III. There were significant differences in disease onset time and urokinase dosage between patients with and without bleeding complications. This indicated that the probability of bleeding increased as the disease onset time and urokinase dosage increased. The differences between age, preoperative NIHSS score and recanalization time between the two groups were not statistically significant.

In addition, the effect of mechanically assisted thrombolysis on bleeding complications was investigated. The results showed that there were 3 cases with bleeding complications and 5 cases without bleeding complications for mechanically assisted thrombolysis, and 6 cases with bleeding complications and 9 cases without bleeding complications for thrombolysis. This indicated that mechanically assisted thrombolysis had no clear effect on bleeding complications.

## Discussion

In this study, the average recanalization time was 65.22 min, and the total recanalization rate was 82.1%, which is relatively higher than that reported in previous studies (11,12,14,17–19). In addition, the mechanically assisted thrombolysis does not increase bleeding complications. This indicates that the recanalization rate and recanalization time of mechanically assisted thrombolysis are more favorable than those of the traditional intravenous and intra-arterial thrombolysis methods. Due to different criteria for patient selection, these data cannot be directly compared with other reported results.

Barreto *et al* have studied the association between thrombus burden (none, mild, moderate and severe, according to thrombus length) and clinical prognosis in 135 patients with acute cerebral infarction. The results showed that for patients with a higher grade of thrombus burden and preoperative NIHSS score, the probability of using mechanically assisted thrombolysis was higher. Following treatment, the recanalization rate was not significantly different from that in patients with a low thrombus burden (20). In this study, the influencing factors on the recanalization rate in mechanically assisted thrombolysis were associated not with the NIHSS preoperative score, but with patient age and disease onset time. The disease onset time is associated with thrombus condition and size, with a clear effect on recanalization rate. The 3 cases with atrial fibrillation were elderly patients. The cerebral infarction may be caused by embolism, but not thrombosis formation, with a poor response to urokinase. Therefore, age may be indirectly associated with the recanalization rate, though with a statistical significance.

In this study, due to mechanical assistance factors, once the vessel was unblocked, the intra-arterial urokinase perfusion could be stopped to minimize bleeding complications. Therefore the urokinase dosage is not associated with the recanalization rate, indicating the importance of mechanically assisted thrombolysis. Therefore, the therapy that acts the most rapidly is significant in obtaining a favorable recanalization rate. Strengthening of public health education on stroke for timely medical treatment and perfection of pre-hospital and in-hospital fast rescue system are the key for successful thrombolytic therapy.

Numerous factors are associated with bleeding complications, including patient age, disease onset time, preoperative NIHSS score, thrombolytic drug dosage, recanalization time, platelet index, blood glucose level and collateral circulation to the infarcted cortex (4,21–26). In this study, only the effects of patient age, disease onset time, preoperative NIHSS score, urokinase dosage and recanalization time on bleeding complication were investigated, due to the small sample size. The results show that there was a significant difference in disease onset time and urokinase dosage between patients with and without bleeding complications. The probability of bleeding increases as the disease onset time and urokinase dosage increase (27). In contrast to previous findings, there is no statistical difference in disease onset age, preoperative NIHSS score and recanalization time between the two groups. This is similar to the findings of by Barreto *et al*(20). In addition, there was no clear effect of mechanically assisted thrombolysis on bleeding complications. The reasons may be that the endovascular surgery increases the vascular endothelial injury, but this side-effect is offset by the decrease of urokinase dosage and thrombolytic time. Therefore the mechanically assisted intra-arterial thrombolysis is safe. Notably, although the bleeding rate in this study is high, the majority of bleedings are ‘imaging bleeding’, but not symptomatic bleeding. This is similar to a previous finding which showed that bleeding patients with recanalization may obtain a favorable prognosis (28). The bleedings may be a leakage of a small volume caused by disruption of the blood-brain barrier.

Stent-assisted revascularization has become an important method in the treatment of acute cerebral infarction (29–34). In the current study, a stent was used in 4 cases for mechanical recanalization, easing vascular stenosis and preventing vascular re-occlusion. In particular, a Solitaire FR stent (EV3, Irvine, CA, USA) was used in one case with a favorable result. However, this still requires further investigation due to the small sample size. In addition, the use of first stent-assisted revascularization is controversial, due to a high postoperative bleeding rate. With the popularization of digital subtraction angiography (DSA) with CT scanning, the intracranial bleeding following thrombolysis may be observed without moving the patient. For patients with combined severe vascular stenosis (without intracranial bleeding), the application of first stent-assisted revascularization may be a good choice for avoiding postoperative vascular re-occlusion.

There are certain deficiencies in this study. Firstly, due to limitations of the mechanical thrombus disruption and retrieval device, saline injection and repetitive interpenetration with a microwire and microcatheter were mainly used in this study, with the occasional use of a stent. With the progress of interventional devices (The Penumbra system; Penumbra, Alameda, CA, USA), the recanalization rate in simple mechanical thrombus disruption and retrieval will be >50% in the near future and thrombolysis is likely to be a complementary therapy for thrombus fragments in distal infarction sites. Secondly, the selected patients in this study were treated within 6 h after disease onset, with abnormal patients excluded by cranial CT scanning. This ensures a short time from the emergency visit to obtaining treatment (<45 min), and thus results in a favorable therapeutic outcome. However, patients with early cerebral edema were excluded from receiving thrombolytic therapy. In addition, the definition of disease onset time is sometimes unclear. The cerebral infarction or progressive aggravation is observed after a patient wakes up. Therefore, applications of clinical-diffusion mismatch, (NIHSS score ≥8, magnetic resonance diffusion abnormalities ≤25 ml) (35), perfusion-diffusion weighted imaging mismatch (36) or CT perfusion scanning (37) in screening and treating patients are more beneficial and require further investigation.

The safety of mechanically assisted thrombolysis for the treatment of acute cerebral infarction is equivalent to that of simple intra-arterial thrombolysis, but the former has a higher efficiency. This method may reduce the urokinase dosage and the recanalization time, and also increase the recanalization rate.

## Figures and Tables

**Table I:** Clinical data of the subjects.

Group	Gender	Age (years)	Onset time (min)	Infarction site score	Preoperative NIHSS score	Postoperative NIHSS score	NIHSS improvement ≥4 in 24 h	NIHSS score (postoperative 3 months)	Mechanical assistance	Thrombolysis time (min)	Urokinase dosage (IU)	Bleeding complication	Postoperative TIMI grade 2-3	mRS score (postoperative 3 months)
1	M	78	290	L-ICA	6	3	No	0	Yes	50	115	No	Yes	Yes
1	M	52	260	Basilar A	25	8	Yes	0	No	60	40	No	Yes	Yes
1	M	59	195	R-ICA	15	8	Yes	2	Yes (stent)	70	100	Yes	Yes	Yes
1	F	66	200	R-M1	21	6	Yes	2	No	55	40	No	Yes	Yes
1	M	61	210	R-M2	9	4	Yes	1	No	50	40	No	Yes	Yes
1	F	78	390	L-M2	12	9	No	4	No	60	75	Yes	Yes	Yes
1	M	54	280	R-M1	12	7	Yes	2	No	90	80	Yes	Yes	Yes
1	M	34	90	R-ICA	7	2	Yes	1	No	75	50	No	Yes	Yes
1	M	51	240	R-M1	8	2	Yes	0	Yes	80	60	No	Yes	Yes
1	F	41	260	R-M1	3	1	No	0	Yes (stent)	70	60	No	Yes	Yes
1	M	59	195	R-ICA	8	2	Yes	0	Yes (stent)	90	100	Yes	Yes	Yes
1	M	57	200	L-M1	11	8	No	2	No	60	60	No	Yes	Yes
1	M	41	210	D-A2	8	6	No	2	No	55	75	No	Yes	Yes
1	F	63	240	L-M2	9	6	No	1	No	60	80	No	Yes	Yes
1	M	36	280	L-M1	12	9	No	3	No	85	80	Yes	Yes	Yes
1	M	61	90	R-A2	3	1	No	0	No	55	50	No	Yes	Yes
1	M	51	240	R-M1	8	2	Yes	0	Yes (stent)	50	60	No	Yes	Yes
1	M	33	360	Right vertebral A	9	5	Yes	3	Yes	60	80	No	Yes	Yes
1	M	59	195	Left vertebral A	15	8	Yes	2	Yes	60	100	Yes	Yes	Yes
1	M	57	200	Right vertebral A	21	6	Yes	2	No	80	55	No	Yes	Yes
1	F	62	210	R-M2	9	4	Yes	1	No	50	40	Yes	Yes	Yes
1	F	64	390	Basilar A	12	9	No	4	No	60	75	Yes	Yes	Yes
1	F	54	280	R-M1	12	7	Yes	2	No	75	80	Yes	Yes	Yes
2	M	57	420	L-ICA	7	5	No	3	Yes	120	45	Yes	No	Yes
2	M	63	450	L-ICA	21	23	No	Died	Yes	60	80	Died	No	No
2	M	62	400	Right vertebral A	10	9	No	5	Yes	60	80	No	No	Yes
2	M	78	390	R-M1	30	20	Yes	Died	Yes	60	80	Died	No	No
2	F	73	380	L-M1	15	18	No	8	Yes	50	80	Yes	No	No

Group 1, recanalization group; Group 2, non-recanalization group. Thrombolysis time was the recanalization time in Group 1. M, male; F, female; NIHSS, National Institutes of Health stroke scale; TIMI, Thrombolysis in Myocardial Infarction; mRS, modified Rankin scale; L-ICA, left internal carotid artery; Basilar A, basilar artery; R-ICA, right internal carotid artery; R-M1, right middle cerebral artery segment 1; R-M2, right middle cerebral artery segment 2; R-A2, right anterior cerebral artery segment 2; D-A2, double anterior cerebral artery segment 2; Right vertebral A, right vertebral artery; Left vertebral A, left vertebral artery.

**Table II:** Indices in patients with recanalization and non-recanalization (mean ± SD).

Indices	Recanalization (n=23)	Non-recanalization (n=5)	P-value
Age (years)	55.26±12.01	66.60±8.62	0.040
Disease onset time (min)	239.35±75.70	408.00±27.75	0.000
Urokinase dosage (U)	69.35±21.65	73.00±15.65	0.725
Preoperative NIHSS score	11.09±5.45	16.60±9.18	0.082

NIHSS, National Institutes of Health stroke scale.

**Table III:** Indices in patients with and without bleeding complications.

Indices	Bleeding (n=14)	No bleeding (n=9)	P-value
Age (years)	53.29±12.74	58.33±11.19	0.340
Disease onset time (min)	220.71±70.22	292.27±88.72	0.034
Urokinase dosage (U)	61.79±20.43	81.11±18.84	0.033
Preoperative NIHSS score	10.57±6.80	11.89±2.31	0.584
Recanalization time (min)	61.43±10.64	71.11±14.74	0.081

NIHSS, National Institutes of Health stroke scale.
